# Supplementary material for: Poverty, quality of life and psychological wellbeing in adults with congenital heart disease in Chile
Source: PLoS One. 2020 Oct 8;15(10):e0240383. doi: 10.1371/journal.pone.0240383 (PMC7544072; doi:10.1371/journal.pone.0240383)
Supplement: Attachment — Submitted filename: Response to Reviewers file.docx [file pone.0240383.s001.docx]

Dear Editor,

I would like to thank you and the reviewers for your valuable comments. This new version has been extensively revised. A major linguistic review was done by a native speaker and other issues were also solved, as commented latter. I am confident that most issues raised by the reviewer were appropriately solved. Specific replies to those comments are stated below.

Regarding the data set issue, I asked the IRB about the feasibility of uploading them to a data based. Unfortunately, they argued that the population is very particular (adult patients suffering from congenital heart diseases and being followed in a particular hospital), hence, even if personal information was deleted, the participants would be easily identifiable. Moreover, the scales used in this study collect information that has been considered highly sensitive by this committee. Therefore, there are ethical reasons that impede the publication of our data set. The agree, however, in that their contact information may be used in case I was not available for researcher interested in this information. The IRB that assessed this study is the Comité Ético Científico de Ciencias de la Salud, its mail is [eticadeinvestigacion@uc.cl](mailto:eticadeinvestigacion@uc.cl) and its phone are (+56) 223548173 / (+56) 22354-2397. I hope this alternative satisfies your requirements.

Kind regards,

Rodrigo López

**Reviewer:**

*Is the manuscript presented in an intelligible fashion and written in standard English? No.*

A major linguistic review was undertaken by a native speaker.

*I appreciate the author's work and dedication to elucidate the relationship among psychological wellbeing and quality of life among adults with complex congenital heart disease.
- The background is thorough and organized well.
- Given extant research is growing and inconclusive, I would like to see this manuscript take a firmer stance on their hypotheses and rationale
- The discussion is well written and comprehensive. Perhaps more of a discussion on the poverty piece (e.g., are patients with CHD more at risk for being in poverty)?
- This manuscript would benefit from a clear paragraph on (1) what this study has added to the literature that exists and (2) what future research should aim to better understand.*

The introduction was reshaped, making clearer the authors’ stance on the topic, what we expected to find and the reasons behind this hypothesis.

The relationship between CHD and poverty was further commented and one new reference was used to illustrate this complex interaction.

One paragraph of the discussion was rephrased to emphasize our contribution to current literature and possible research projects to advance in the knowledge of this issue.
